# Imaging of HER2 may improve the outcome of external irradiation therapy for prostate cancer patients

**DOI:** 10.3892/ol.2014.2760

**Published:** 2014-12-03

**Authors:** JENNIE ANDERSSON, MARIA ROSESTEDT, ANNA ORLOVA

**Affiliations:** Department of Medicinal Chemistry, Preclinical PET Platform, Uppsala University, Uppsala 751 83, Sweden

**Keywords:** prostate cancer, external irradiation, molecular imaging, human epidermal growth factor receptor type 2

## Abstract

Prostate cancer (PCa) is the most common type of cancer among males. Human epidermal growth factor receptor type 2 (HER2) expression in PCa has been reported by several studies and its involvement in the progression towards androgen-independent PCa has been discussed. External irradiation is one of the existing therapies, which has been demonstrated to be efficient in combination with androgen deprivation therapy for the treatment of advanced PCa. However, 20–40% of patients develop recurrent and more aggressive PCa within 10 years. The current study investigates the involvement of HER2 in survival and radioresistance in PCa cells and we hypothesized that, by monitoring HER2 expression, treatment may be personalized. The PCa cell lines, LNCap, PC3 and DU-145, received a 6 Gy single dose of external irradiation. The number of PC3 cells was not affected by a single dose of radiation, whereas a 5-fold decrease in cell number was detected in LNCap (P<0.00001) and DU-145 (P<0.0001) cells. The HER2 expression in PC3 exhibited a significant increase post irradiation, however, the expression was stable in the remaining cell lines. The administration of trastuzumab post-irradiation resulted in a 2-fold decrease in the PC3 cell number, while the drug did not demonstrate additional effects in LNCap and DU-145 cells, when compared with that of irradiation treatment alone. The results of the present study demonstrated that an increase in membranous HER2 expression in response to external irradiation may indicate cell radioresistance. Furthermore, imaging of HER2 expression prior to and following external irradiation may present a step towards personalized therapy in PCa.

## Introduction

Prostate cancer (PCa) is the most common type of cancer in males. This cancer type has a heterogeneous nature and the characteristics vary during development. Initially, PCa develops in the prostate gland and is dependent on the androgen, testosterone for proliferation, growing slowly. At present, no optimal treatment has been identified due to the difficulties of predicting the disease progression (1,2). However, treatment of PCa is not always required, and the most common course of action is referred to as ‘watchful waiting’. In total, ~30% of patients on watchful waiting begin an active treatment within the first five years following diagnosis; ~66% of these patients undergo a radical prostatectomy and ~20% receive external irradiation (2). However, the applied treatment is guided by the tumor characteristics and the health status and age of the patient. When the tumor is no longer localized to the prostate gland and begins to invade surrounding healthy tissue, the applied treatment is more urgent and aggressive. The preferable treatment at this stage is a combination of androgen ablation therapy and local irradiation (3), which results in a clinically stable state for the patient, which lasts for 1.5–3 years (4).

External irradiation is a common treatment for PCa, and novel treatment regimens have been developed in order to increase the doses received by the tumor, whilst sparing the surrounding tissues. By using image-guided radiation therapy or intensity modulated therapy, doses of 78–81 Gy may be administered while the healthy tissue surrounding the tumor is spared. Combinations with selected radiation boost regimens such as brachytherapy, may achieve doses of >116 Gy (5). However, 20–40% of patients receiving external irradiation therapy develop recurrent and more aggressive PCa within 10 years (6). The absence of androgen contributes to the clonal selection of androgen independent cells. This generates a tumor with an altered phenotype, which is more aggressive, less responsive to existing therapies and exhibits a higher metastasizing potential (7). Human epidermal growth factor receptor type 2 (HER2) is a receptor tyrosine kinase (RTK), which has been identified in 17–22% of analyzed PCa tissues (depending on the antibody used for detection) in a large retrospective study (8). The function of HER2 in an androgen diminished environment is considered to promote cell division and suppress apoptosis and thus, the protein expression is significantly associated with a more advanced disease, tumor stage and recurrence (9). HER2 is an essential factor in one of the pathways that allows PCa cells to survive and proliferate, resulting in the development of androgen-independent metastatic PCa (10). The overexpression of HER2 in PCa has the capacity to activate androgen receptors in the absence of androgens, as well as promote the transcription of prostate specific antigen (11,12). As discussed, PCa may relapse following external irradiation treatment and HER2-expressing cells are hypothesized to activate survival mechanisms as a response to the treatment, which contributes to higher proliferation and reduced apoptosis rates (13). This enables the selection of HER2-expressing cell subpopulations, leading to a progression towards androgen-independence (14). At present, trastuzumab (Herceptin) is used clinically for the treatment of HER2-expressing breast cancer (BCa) and studies, including the use of trastuzumab in bladder (15), endometrial (16), peritoneal, ovarian, pancreatic and stomach neoplasms (17) have been previously reported. In the current study, the suitability of HER2 as a target for treatment of PCa alone or in combination with external irradiation was investigated, and the effect of trastuzumab on PCa cell survival was analyzed. In addition, patient stratification and therapy outcome were hypothesized to be significantly influenced by accurate molecular phenotyping, which may indicate suitable molecular targets, and lead to the development of appropriate imaging agents. We further hypothesized that *in vivo* molecular imaging of HER2 expression in PCa may contribute to an improved patient selection, as well as improved therapy outcomes.

The specific aims of this study were to analyze and evaluate PCa cell survival, as well as the HER2-expression as an acute response to external irradiation and anti-HER2 drug treatment, such as trastuzumab. In total, three cell lines, LNCap (lymph node metastasis of PCa, androgen and estrogen receptor positive), PC3 (bone metastasis of PCa, androgen sensitive) and DU-145 (brain metastasis of PCa, hormone insensitive) were selected for this study. Together, these cell lines may represent the tumor heterogeneity due to differences in androgen sensitivity and aggressiveness. The cell panel was treated with external irradiation, modeling one of the current therapy modalities for a localized disease, alone or in combination with a HER2-targeting drug. Cell survival, as well as membranous expression of HER2 in response to therapy, was investigated. Trastuzumab, a clinically approved therapeutic monoclonal antibody (Herceptin), which binds to the extracellular domain of HER2 and downregulates its expression (18), was selected for this study.

## Materials and methods

### Cell lines and treatment

The cell lines LNCap, PC3 and DU-145 originally from the American Type Culture Collection (Manassas, VA, USA) were provided by LGC Standards (Borås, Sweden). The HER2-receptor expression of the cell lines was evaluated in a previous study (19). The cells were cultured in complete RPMI-media, supplemented with 10% fetal bovine serum, 2 mM L-glutamate, 100 IU/ml penicillin and 100 μg/ml streptomycin. For LNCap cells, the medium was supplemented with sodium-pyruvate (Lonza, Verviers, Belgium) and HEPES. All other reagents including trypsin-EDTA were obtained from Biochrom AG Biotechnologie (Berlin, Germany). All plastics for cell culturing were obtained from Corning, Inc. (Corning, NY, USA) for cell cultivation. Cell culture was performed in a humidified atmosphere of 5% CO_2_ at 37°C.

Trastuzumab (infusion, 21 mg/ml) was used for *in vitro* treatment. The drug was diluted in cell cultivation medium to 0.05 mg/ml. External irradiation was performed using a Gammacell 40 Exactor (^137^Cs γ-ray photon radiation; Nordion, Ottawa, ON, Canada).

For HER2 quantification the affibody molecule, Z_2395_ (Affibody AB, Solna, Sweden), was used. Radiolabeling of Z_2395_ with technetium-99m was performed as described by Ahlgren *et al* (20). Radioactivity was measured using an automated γ-counter with a 3-inch NaI (Tl) detector (1480 WIZARD; PerkinElmer Life Sciences, Waltham, MA, USA). Cells were counted using an electronic Scepter™ cell counter (Millipore, Billerica, MA, USA).

### Statistical analysis

Student’s *t*-test was used to evaluate the significance of changes in proliferation and receptor expression. ^*^P<0.05 was considered to indicate a statistically significant difference.

### External irradiation and drug treatment

**Figure 1:**
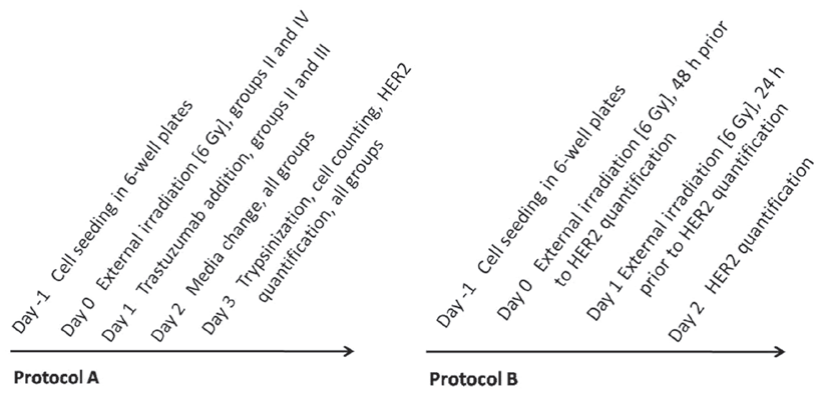
Treatment protocols for PCa cell lines. Protocol A: Treatment for groups I-IV, which consisted of a single 6 Gy dose of external irradiation, alone (group II) or in combination with 0.05 mg/ml trastuzumab administered post irradiation (group IV). One group received 0.05 mg/ml trastuzumab alone (group III). A control group (I) was maintained under the same conditions, without exposure to any treatments. Protocol B: Treatment for PCa cell lines receiving external irradiation alone. Receptor quantification was performed 24 or 48 h following irradiation exposure. PCa, prostate cancer.

Cells were treated according to protocol A (Fig. 1). Cells were seeded at a density of 10^6^ cells/well in six-well plates one day prior to the experiments. The cells were subjected to a 6 Gy dose of external irradiation (group II), treatment with trastuzumab (group III), or a combination of the two (group IV). One group of cells was used as a control (group I) and treated in the same manner as all other cells, without exposure to any drug or irradiation. All experiments were performed in triplicate.

### Receptor quantification

Quantification of HER2 expression was conducted 24 and 48 h post irradiation exposure in all groups (I-IV). To evaluate the changes in receptor expression as response to external irradiation alone, cells were seeded as described, one day prior to the first irradiation exposure and treated according to protocol B (Fig. 1). For the experiment, 100 nM of unlabeled Z_2395_ was added to half of the dishes and the cells were subsequently incubated for 1 h at room temperature. This was followed by the addition of 10 nM ^99m^Tc-labeled Z_2395_ to all dishes and incubation for 1 h. The cells were subsequently trypsinized, resuspended, collected and counted according to protocols described previously (19,21). The radioactivity in cell samples was measured using an automated γ-counter. All experiments were performed in triplicate.

## Results

**Figure 2:**
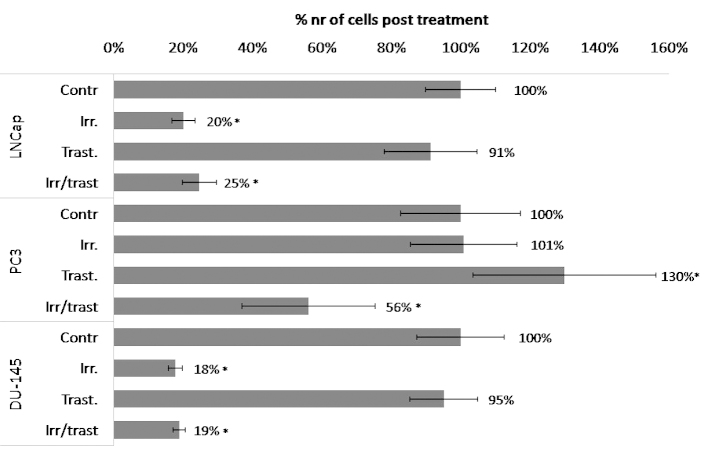
Changes in proliferation rate in response to treatment. The graph shows changes in cell proliferation following treatment with irradiation/trastuzumab, which was administered in accordance with protocol A. The results are presented as the percentage change in proliferation (number of cells) compared with that of the control. Significance is indicated in the graph by ^*^ and standard deviation is indicated by the error bars.

**Figure 3:**
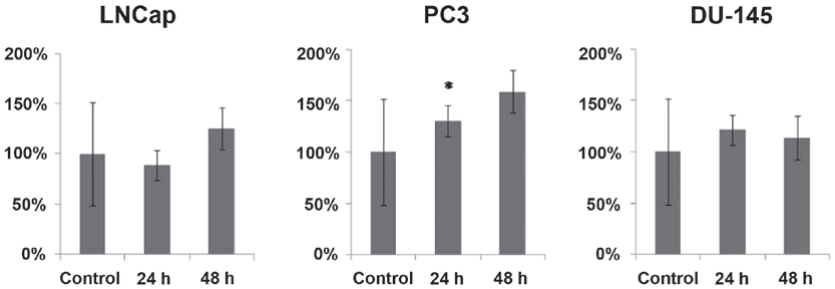
Changes in HER2 expression following irradiation in PCa cell lines. The graphs shows changes in HER2 expression in PCa cell lines following a single 6 Gy dose of external irradiation. The results are shown as the percentage change in HER2 expression (per cell) compared with that of the control. Significance is indicated in the graph by ^*^ and standard deviation is indicated by the error bars. PCa, prostate cancer; HER2, human epidermal growth factor receptor type 2.

PCa cell lines treated with a 6 Gy dose of external irradiation exhibited a varied response with regards to cell survival and HER2 expression. Irradiation treatment alone was not observed to exert any effect on PC3 cells, whereas a 5-fold decrease in cell survival was detected in the LNCap (P<0.00001) and DU-145 (P<0.0001) cells (Fig. 2). After 24 h, PC3 cells exhibited a significant 30% increase in HER2-expression and 48 h post-irradiation, the receptor expression was 50% higher than that of the untreated control cells (Fig. 3). No similar response was detected in the other cell lines, which maintained stable receptor expression.

The HER2-targeting drug, trastuzumab, was administered following irradiation treatment to evaluate its potential additive effect (Fig. 2). The PC3 cells that received trastuzumab treatment only, exhibited a significant increase in cell number, however, the post-irradiation administration of trastuzumab resulted in a 2-fold decrease in cell number when compared with the untreated control cells and a 2.5-fold decrease when compared with that of trastuzumab alone. No additive effect was identified in LNCap and DU-145 cells, when compared with irradiation treatment alone.

## Discussion

The most common first line treatment for disseminated PCa is external irradiation, which is often combined with removal or blockage of androgen. However, 20–40% of patients relapse within 10 years following treatment. The occurrence of relapsing and more aggressive PCa with androgen independent clones is considered to be caused by an activation of androgen-independent pathways, required for cancer progression (11). HER2 is a RTK, which is known to be upregulated in numerous types of cancer, promoting cell motility, division and suppressing apoptosis. Previous studies have consistently reported HER2 expression in PCa (8,22) as well as its clear involvement in the androgen independent PCa (23). The pathway that allows continued PCa cell proliferation is dependent on HER2, and overexpression of the protein may activate androgen receptors in the absence of androgen (9). A number of arguments have been presented for the utilization of HER2 as a potential target for the treatment of PCa. We hypothesize that HER2 may be involved in radioresistance and measuring of HER2-expression as a response to external irradiation may be used to monitor treatment response in PCa for the stratification of patients and their response to additional therapy. We further hypothesized that anti-HER2 therapy shortly following irradiation treatment may overcome radioresistance.

In the current study, a panel of PCa cell lines was used to represent the heterogeneity of the disease. As the tumor is not homogenous, the use of one cell line would not adequately represent the various characteristics of PCa. Therefore, three PCa cell lines, LNCap, PC3 and DU-145, were selected, which exhibit different metastatic potentials and proliferation rates, as well as different degrees of androgen independence. Together the cell lines constitute a broad panel for preclinical PCa studies.

The level of HER2 expression in the selected cell lines is considered to be 20,000–50,000 receptors/cell (19) which correlates with clinical data regarding HER2 expression in PCa and corresponds to the very low expression in BCa (+1 according to a HercepTest), where tumor biopsies with a score of 1+ (a faint/barely perceptible membrane staining in >10% of tumor cells) are considered to present negative HER2-expression, however, a score of 3+ (strong complete membrane staining is observed in >10% of tumor cells) is considered to present strong positive HER2-expression (8). However, the detection and visualization of such low HER2 expression levels is now possible with highly sensitive small protein-based imaging probes, such as affibody molecules (19,24,25).

In this study, PCa cells were exposed to one dose of external radiation (6 Gy), which corresponds to one dose of fractionated therapy, and the short-term response in cell survival and HER2 expression was analyzed as a model for early therapy modeling. The rapid response to this treatment was observed as a 5-fold reduction in cell number in LNCap and DU-145 cells when compared with untreated controls (Fig. 2), whereas PC3 cells demonstrated radioresistance. The HER2 expression in LNCap and DU-145 cells remained stable 48 h following irradiation, whereas the receptor expression in PC3 cells significantly increased by 50% after 48 h (Fig. 3). As the HER-family is involved in cell survival and proliferation, the increase in receptor expression observed is consistent with the activation of this cell-survival mechanism. This protective behavior is typical for HER2 expressing cells and has been demonstrated previously (26). Therefore, we hypothesize that these results, which showed an increase in the membranous expression of HER2 as an acute response to external irradiation, indicate cell radioresistance.

In order to investigate whether the HER2 targeting drug, trastuzumab, exhibits an effect post irradiation, PCa cells were exposed to trastuzumab treatment following external irradiation, or to trastuzumab treatment alone. Treatment with trastuzumab alone did not exhibit an inhibitory effect on cell survival, and notably demonstrated a pro-proliferative effect on PC3 cells (Fig. 2). Although PC3 cells were not affected by irradiation or anti-HER2 treatment, the trastuzumab administration post-irradiation was efficacious, and resulted in a significant 44% decrease in cell number. These results are also consistent with those presented in another study, investigating the effect of irradiation on HER2 positive and negative BCa cell lines, which received HER2 targeting therapy (27). The results of the present study may indicate a suppressive action of trastuzumab on the survival mechanism mediated by HER2 expressing cells. DU-145 and LNCap cells exhibited radiosensitivity, however, no additive effects were observed following the post-irradiation administration of trastuzumab.

This study preclinically evaluated a combination of clinically available therapies and drugs, and their effect on cell survival and HER2 expression. External irradiation therapy in combination with HER2-targeting drugs was demonstrated to alter the receptor expression profile in radioresistant PCa cell lines. The observed changes in HER2 expression as a response to this treatment regime may be used to monitor the response to therapy.

In conclusion, measurement of HER2 expression prior to and following therapy may present a step towards patient stratification and more personalized treatment, as well as emphasizing the potential requirement for additional therapy. Additional studies investigating how analysis of HER2 in a xenograft model may serve as a marker to identify non-responders that may benefit from other treatments are required.
